# A Mixture of Soybean Oil and Lard Alleviates Postpartum Cognitive Impairment via Regulating the Brain Fatty Acid Composition and SCFA/ERK(1/2)/CREB/BDNF Pathway

**DOI:** 10.3390/nu16162641

**Published:** 2024-08-10

**Authors:** Runjia Shi, Xiaoying Tian, Andong Ji, Tianyu Zhang, Huina Xu, Zhongshi Qi, Liying Zhou, Chunhui Zhao, Duo Li

**Affiliations:** 1 Institute of Nutrition and Health, Qingdao University, Qingdao 266071, China; srjcharacter@163.com (R.S.); jiandongqd@163.com (A.J.); zhangty930902@126.com (T.Z.); xuhuina1998@163.com (H.X.); qizhongshi0414@163.com (Z.Q.); zhouly90722@163.com (L.Z.); zch174199@163.com (C.Z.); 2 School of Public Health, Qingdao University, Qingdao 266071, China; 3 Qingdao Medical College, Qingdao University, Qingdao 266071, China; 18363972898@163.com

**Keywords:** mixture of soybean oil and lard, *n*-3 polyunsaturated fatty acids, lipid nutrition, postpartum cognitive function

## Abstract

Lard is highly appreciated for its flavor. However, it has not been elucidated how to consume lard while at the same time eliminating its adverse effects on postpartum cognitive function. Female mice were divided into three groups (*n* = 10): soybean oil (SO), lard oil (LO), and a mixture of soybean oil and lard at a ratio of 1:1 (LS). No significant difference was observed between the SO and LS groups in behavioral testing of the maternal mice, but the LO group was significantly worse compared with these two groups. Moreover, the SO and LS supplementation increased docosahexaenoic acid (DHA) and total *n*-3 polyunsaturated fatty acid (PUFA) levels in the brain and short-chain fatty acid (SCFA)-producing bacteria in feces, thereby mitigating neuroinflammation and lowering the p-ERK(1/2)/ERK(1/2), p-CREB/CREB, and BDNF levels in the brain compared to the LO group. Collectively, the LS group inhibited postpartum cognitive impairment by regulating the brain fatty acid composition, neuroinflammation, gut microbiota, and the SCFA/ERK(1/2)/CREB/BDNF signaling pathway compared to lard.

## 1. Introduction

During the stages of pregnancy, childbirth, and lactation, there occurs a multifaceted reorganization encompassing physiological, psychological, and socio-environmental dimensions [1]. Concurrently, the maternal brain experiences notable structural and functional neuroplasticity, alongside cognitive adaptations throughout the peripartum duration [2,3]. The epidemiological study revealed that a minimum of 20% of women were susceptible to cognitive impairment during pregnancy, postpartum, or across both intervals [4]. The dietary consumption of lipids and fatty acids is closely linked to cognition function, highlighting the necessity of assessing how dietary oils impact postpartum cognitive function.

As a traditional dietary oil, lard has been cherished for its unique flavor and taste for thousands of years in China [5]. But previous research indicated that lard exerted a detrimental effect on cognitive function. For example, rats fed a high-fat lard diet exhibited inferior performance during an open-field test [6]. In addition, after more than 8 weeks on a high-fat lard and high-sucrose diet, male rats demonstrated behaviors suggesting anxiety [7]. Therefore, how to consume lard while mitigating its adverse effects on postpartum cognitive function is a pressing issue that needs to be addressed. Interestingly, our previous randomized clinical trials have demonstrated that the mixture of soybean oil (SO) and lard oil (LO) at a ratio of 1:1 may confer greater benefits on cardiometabolic risk factors and liver function compared with either soybean oil or lard alone [8,9]. Thus, we hypothesized that the consumption of the mixture of soybean oil and lard at the ratio of 1:1 (LS) had a preventive effect on cognitive impairment in postpartum mice compared to lard.

The significance of the gut–brain axis in maintaining central nervous system functions has been recognized for an extended period. Alterations in this signaling pathway may contribute to a range of central nervous system dysfunctions, spanning from neurodevelopmental disorders to neurodegenerative diseases [10]. Accumulating evidence suggested a potential pivotal role of short-chain fatty acids (SCFAs) a microbial metabolites, in mediating gut-brain axis signaling [11]. For example, fish oil has been reported to improve neuropsychiatric behaviors by increasing SCFA levels [12]. The lard group exhibited lower concentrations of total SCFAs, acetic acid, and propionic acid compared to the soybean oil and fish oil groups in both cecal and colonic contents [13]. And a previous study showed that prolonged acetate deficiency led to a decrease in hippocampal recombinant synaptophysin levels and expedited cognitive decline in mice [14]. In addition, SCFAs have been demonstrated to activate the cyclic adenosine monophosphate (cAMP) pathway, including potentiating the phosphorylation of extracellular signal-regulated kinases 1 and 2 (ERK1/2), along with their downstream responses within this pathway [15,16]. Of note, increasing evidence suggested that upregulating the expression of the ERK(1/2)/cyclic AMP response element-binding protein (CREB)/brain-derived neurotrophic factor (BDNF) pathway-related proteins may ameliorate neuronal damage, thereby alleviating induced cognitive impairments [17,18]. Prior research has revealed that mumefural treatment ameliorated cognitive impairment by regulating p-ERK/ERK, p-CREB/CREB, and BDNF levels [19]. However, it is unclear whether SO, LO, and LS have similar effects via the SCFA/ERK(1/2)/CREB/BDNF signaling pathway in the postpartum mouse brain.

To our knowledge, there has been no research on the effects of SO, LO, and LS interventions during the pre-pregnancy to postpartum period on the cognitive function of maternal mice. Here, the neuroimmune statuses of the maternal mice were investigated. Subsequently, the brain fatty acid profiles, the gut microbiota compositions, the SCFA contents, and the levels of ERK(1/2)/CREB/BDNF signaling pathway-related proteins were performed.

## 2. Materials and Methods

### 2.1. Animals and Study Design

**Figure 1:**
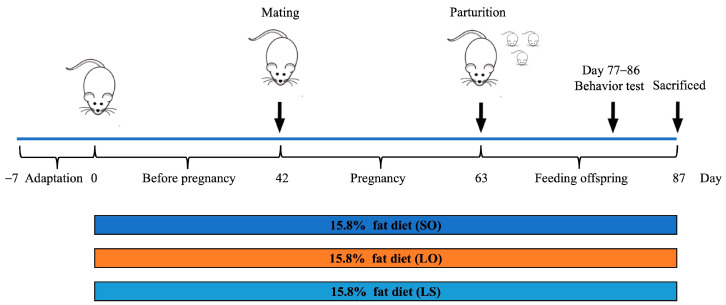
The schematic depicting the present study design.

Female C57BL/6JNifdc mice aged six weeks and male C57BL/6JNifdc mice aged seven weeks were sourced from Beijing Charles River Laboratory Animal Technology Co., Ltd. (Beijing, China). All mice were housed under controlled conditions: temperature maintained at 22 ± 1 °C, humidity at 50 ± 5%, and a 12 h light/12 h dark cycle, with ad libitum access to food and water. The experimental design flowchart is illustrated in Figure 1. After a week acclimatization, the female mice were randomly assigned into three groups (*n* = 10): SO group, LO group, and LS group and provided with diets containing identical fat content but varying in fat types (15.8% energy from fat), sourced from Jiangsu Xietong Pharmaceutical Bio-engineering Co., Ltd. (Jiangsu, China). The ingredients and fatty acid composition of three diets are detailed in Tables S1 and S2, respectively. After feeding for 6 weeks, the female and male mice were housed together in cages, and the presence of female vaginal plugs was examined the following morning; if detected, that day was recorded as the successful day of pregnancy. The diet of the maternal mice remained consistent throughout the gestation and lactation periods. Following behavioral assessments, all mice were fasted overnight before being sacrificed. Brain samples were stored at −80 °C until subsequent analysis. The experimental protocols were conducted in line with the Guidelines for Care and Use of Laboratory Animals of Qingdao University. The Ethics Committee of the Medical College of Qingdao University (No. QDU-AEC-2024403) approved this study.

### 2.2. Behavioral Tests

The open-field test for spontaneous activity and anxiety, the Y-maze test for working memory, and the Morris water maze test for spatial learning and memory, were conducted on the maternal mice. During the open-field test, mice freely explored a 40 × 40 × 40 cm box for 5 min, while in the Y-maze test, they freely explored three equally long arms for 5 min. The Morris water maze test utilized a circular pool filled with TiO_2_-mixed water. The operational procedures followed previous research protocols [20].

### 2.3. The Staining of Hematoxylin and Eosin (H&E) and Nissl

The total brain samples were fixed in 4% paraformaldehyde and embedded in paraffin. H&E staining procedure: sections were sequentially treated with de-waxing solutions, absolute ethanol, 75% ethanol, and rinsed with water before fixation. They were stained with hematoxylin, differentiated, blued, and dehydrated with eosin staining. Sections were then cleared in absolute ethanol and xylene and mounted with neutral gum. Nissl staining procedure: sections were sequentially treated with xylene, absolute ethanol, 75% ethanol, rinsed with water, stained with aniline blue, rinsed again, dried at 60 °C, cleared in xylene, and mounted with neutral gum. Imaging of all sections was conducted using a NIKON DS-U3 microscope (Tokyo, Japan).

### 2.4. Analysis of Fatty Acid Composition

Briefly, 50 mg of brain tissue was transferred to glass tubes and mixed with a solution of methanol/chloroform (1:1). Subsequently, after methyl esterification treatment, the samples were filtered and analyzed using gas chromatography (Agilent 7890A, Agilent Technologies, Santa Clara, CA, USA).

### 2.5. Inflammatory Cytokines Analysis

A total of 10 mg brain tissue was homogenized in 100 μL precooled saline and subsequently centrifuged at 3000 rpm for 10 min at 4 °C. The levels of inflammatory cytokines, including TNF-α (JM-02415M2), IL-6 (JM-02446M2), IL-1β (JM-02323M2), and IL-18 (JM-02452M2) were quantified using ELISA kits (Jiangsu Jingmei Biotechnology Co., Ltd., Yancheng, Jiangsu, China) following the manufacturer’s protocol.

### 2.6. Immunofluorescence Staining

Whole-brain samples were utilized for immunofluorescent staining targeting glial fibrillary acidic protein (GFAP, 1:1000, Servicebio, Wuhan, China) and ionized calcium-binding adapter molecule 1 (IBA1, 1:1000, Servicebio, Wuhan, China). Briefly, the deparaffinized and rehydrated tissue sections underwent antigen retrieval using EDTA solution. All sections blocked were incubated with primary antibodies (1:500, Servicebio, Wuhan, China) against GFAP and IBA1. Finally, all sections were incubated with secondary antibodies for 50 min following washing with PBS.

### 2.7. Western Blotting

In brief, the 30 mg brain samples were lysed using 300 μL RIPA lysis buffer to extract proteins. The homogenates were then centrifuged (14,000× *g*, 10 min, 4 °C), and the supernatants containing total protein were collected and quantified the protein concentrations by the BCA protein assay kits (Beyotime, Beijing, China). Equal amounts of proteins (15–30 μg per lane) were separated using 8–12% SDS-PAGE and transferred onto PVDF membranes (Merck Millipore, Billerica, MA, USA). Following blocking with TBST, membranes were washed three times and subsequently incubated with specific primary antibodies, viz. NLRP3, ASC, cleaved caspse-1, p-CREB, and CREB from Affinity with dilution ratios 1:1000 and p-ERK(1/2), ERK(1/2), BDNF, and PSD-95 from Abcam (Cambridge, UK) with dilution ratios 1:1000. Subsequent to three additional TBST washes, the membranes were incubated by specific secondary antibodies. Finally, band detection was performed using a Fusion Solo S (Vilber Lourmat, Fontenay-sous-Bois, France).

### 2.8. Gut Microbiota Analysis

The fecal samples of the maternal mice were submitted to Biomarker Technologies Co., Ltd. (Beijing, China) for 16S rRNA detection. The TGuide S96 Magnetic Soil/Stool DNA Kit (Tiangen Biotech (Beijing) Co., Ltd, Beijing, China) was used to extract total genomic DNA from all samples. The V3-V4 region of the 16S rRNA gene was amplified using universal primers. After purifying, quantifying, and homogenizing PCR products, sequencing libraries were prepared, followed by paired-end sequencing conducted on an Illumina NovaSeq6000 platform (Illumina, Santiago CA, USA). After denoising sequences with 97% similarity, operational taxonomic units (OTUs) were clustered using USEARCH (version 10.0). Taxonomic classification of all OTUs was performed by searching against the Silva databases using QIIME software (2020.6.0).

### 2.9. Detection of SCFAs in Feces

In short, 100 mg of fecal samples was mixed with 1 mL of ultrapure water, followed by the addition of sulfuric acid and internal standard solution (cyclohexanone, Sigma-Aldrich, St. Louis, MO, USA). After addition of 1 mL of ether and homogenization for 1 min, the mixture was centrifuged and the supernatant was analyzed using gas chromatography–mass spectrometry (8890-7693A, Agilent Technologies, Santa Clara, CA, USA).

### 2.10. The Transmission Electron Micrograph in the Hippocampus

The sliced 1 mm^2^ hippocampal tissue blocks were fixed with 2.5% glutaraldehyde. After washing with PBS, the tissues were incubated in 1% osmium tetroxide (Ted Pella Inc., Redding, CA, USA) in the dark for 2 h. Following another three washes with 0.1 M PBS, the tissues were dehydrated sequentially in ethanol and acetone. After embedding and polymerization, the resin blocks were sectioned into 60–80 μm slices, followed by staining with uranyl acetate-saturated alcohol solution (SPI Supplies, West Chester, PA, USA) and lead citrate solution. All sections were analyzed by a transmission electron microscope (HITACHI, Tokyo, Japan).

### 2.11. Statistical Analysis

The data are reported as mean ± SD or median (interquartile range). Group differences were assessed using non-parametric tests or one-way ANOVA, with subsequent post hoc Tukey tests to determine significance. The correlations between behavioral testing outcome indicators and brain fatty acids or gut microbiota were evaluated by Pearson correlation analysis. The statistical analyses were implemented by GraphPad Prism version 10.2.3 (GraphPad Software, San Diego, CA, USA). The potential mechanism was visually depicted with a schematic diagram created using Figdraw.

## 3. Results

### 3.1. Effect of a Mixture of Soybean Oil and Lard on Postpartum Cognitive Function

**Figure 2:**
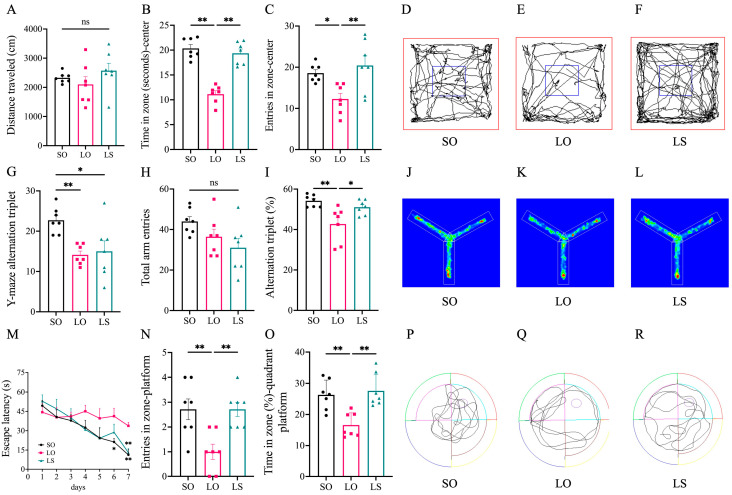
Effect of a mixture of soybean oil and lard on postpartum cognitive function. (**A**–**C**) The detection results and (**D**–**F**) the representative traveled path of maternal mice in the open-field test. (**G**–**I**) The detection results and (**J**–**L**) the representative traveled path of maternal mice in the Y-maze test. (**M**–**O**) The detection results and (**P**–**R**) the representative traveled path of maternal mice in the Morris water maze test. Data represent mean ± SD (*n* = 7). * *p* < 0.05 and ** *p* < 0.01 represent the significant difference.

No significant differences were observed in the distance traveled during the open-field test among the three groups of mice. Compared with the SO group, the LO group exhibited significantly lower values in terms of time in zone (seconds)–center and entries in zone–center, and the SO group and LS group showed comparable results with no significant difference observed between them. (Figure 2A–F). Furthermore, the LO group of maternal mice showed a significantly lower alternation triplet (%) than the SO group, and no significant difference was observed between the SO group and the LS group in the Y-maze test (Figure 2G–L). The results of the Morris water maze test (Figure 2M–R) show that all three groups of maternal mice showed improved performance in locating the platform position after each day of training. During the probe test of the seventh day, the LO group of mice exhibited significantly higher escape latency (s) and lower entries in zone-platform and time in zone (%)-quadrant platform than the SO group. The SO group showed no significant difference compared with the LS group.

### 3.2. Effect of a Mixture of Soybean Oil and Lard on Brain Fatty Acid Composition and Its Correlation with Behavioral Testing Outcome Indicators

**Figure 3:**
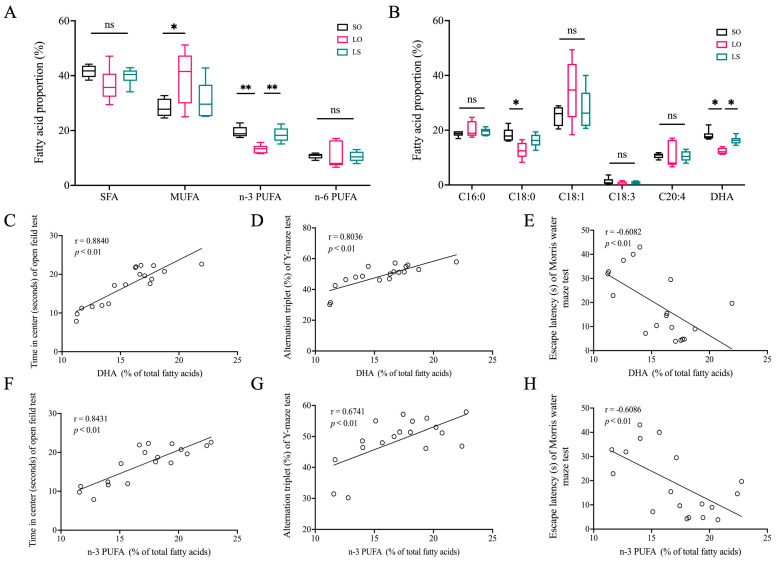
Effect of a mixture of soybean oil and lard on brain fatty acid profile and its correlation with behavioral testing outcome indicators. (**A**,**B**) Brain fatty acid proportions (%). (**C**–**H**) Pearson’ correlations between DHA and *n*-3 PUFA levels in the maternal mice brain and behavioral testing outcome indicators. Data represent median (interquartile range) (*n* = 6). * *p* < 0.05 and ** *p* < 0.01 represent the significant difference.

The gas chromatography analysis of the brain fatty acid composition in the postpartum mice (Table 1 and Figure 3) revealed no significant differences (*p* > 0.05) in Saturated fatty acid (SFA) and *n*-6 polyunsaturated fatty acid (PUFA) levels among the three groups. However, the *n*-3 PUFA level in the LO group was significantly lower compared with both the SO (*p* = 0.0004) and LS (*p* = 0.002) groups. Moreover, the LO group exhibited a significantly higher content (*p* = 0.0373) of monounsaturated fatty acid (MUFA) than the SO group. Additionally, compared with the SO (*p* < 0.0001) and LS (*p* = 0.0012) groups, the LO group showed significantly lower levels of DHA, while the level of C18:0 was significantly lower only compared with the SO (*p* = 0.005) group. Interestingly, the levels of DHA and *n*-3 PUFA in the maternal mice brain were significantly positively correlated with time in center (seconds) and alternation triplet (%), while showing a significant negative correlation with escape latency (seconds).

### 3.3. Effect of a Mixture of Soybean Oil and Lard on Brain Histopathology and the Activation of Neuroglial Cells

**Figure 4:**
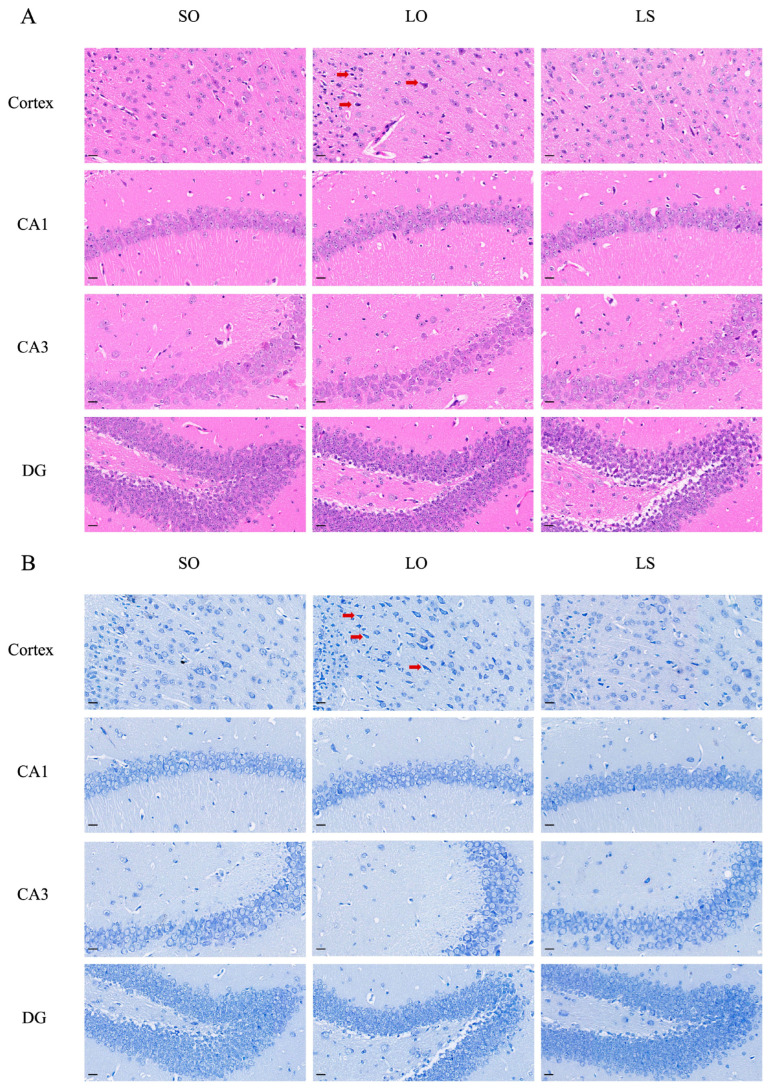
Effect of a mixture of soybean oil and lard on brain histopathology. (**A**) The H&E and (**B**) Nissl staining diagrams in the brain (scale bar = 20 μm). Red arrows: nuclei pyknosis.

**Figure 5:**
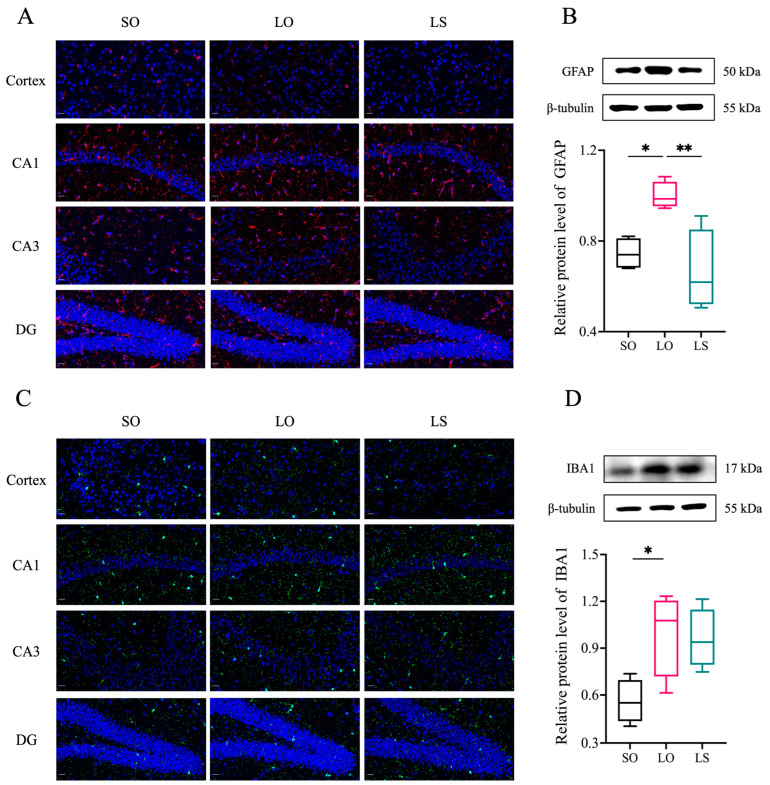
Effect of a mixture of soybean oil and lard on the activation of neuroglial cells. Brain GFAP expression was analyzed via (**A**) immunofluorescence and (**B**) Western blotting (*n* = 4). IBA1 expression in the brain was analyzed via (**C**) immunofluorescence and (**D**) Western blotting (*n* = 4). Scale bar = 20 μm. Data represent median (interquartile range). * *p* < 0.05 and ** *p* < 0.01 represent the significant difference.

Histological staining (H&E and Nissl) in Figure 4 revealed nuclei pyknosis (red arrows) in the cortex of the LO group, along with a reduced proportion of normal neurons compared with the SO and LS groups. Interestingly, no noticeable differences were observed among the three groups in the hippocampal circuit regions (CA1 and CA3) and dentate gyrus (DG). Immunofluorescence analysis of the GFAP and IBA1 levels in the cortex and hippocampus (Figure 5A,C) corroborated these findings, showing increased astrocyte and microglia activity in the cortex of the LO group compared with the SO and LS groups. Furthermore, Western blotting demonstrated significantly higher levels of GFAP and IBA1 expression in the LO group compared with both the SO and LS groups (Figure 5B,D).

### 3.4. Effect of a Mixture of Soybean Oil and Lard on Brain Neuroinflammation

**Figure 6:**
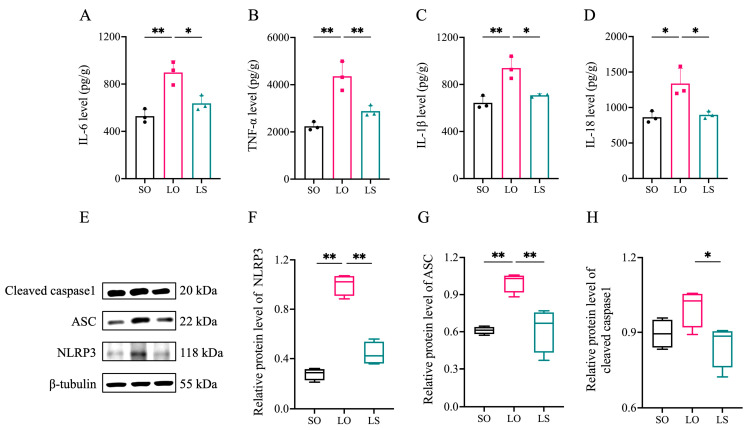
Effect of a mixture of soybean oil and lard on brain neuroinflammation. The levels of (**A**–**D**) inflammatory cytokines (pg/g, *n* = 3) and (**E**–**H**) NLRP3 inflammasome complex-related proteins (*n* = 4). Data represent mean ± SD or median (interquartile range). * *p* < 0.05 and ** *p* < 0.01 represent the significant difference.

The LO group exhibited significantly higher levels of IL-6, TNF-α, IL-1β, and IL-18 in the brain compared with both the SO and LS groups (Figure 6A–D). Moreover, NLRP3 inflammasome complex-related proteins, including NLRP3 and ASC, were significantly higher in the LO group compared with the SO and LS groups. Interestingly, the level of cleaved caspase-1 in the LO group was only significantly higher than that observed in the LS group.

### 3.5. Effect of a Mixture of Soybean Oil and Lard on the Gut Microbiota Composition

**Figure 7:**
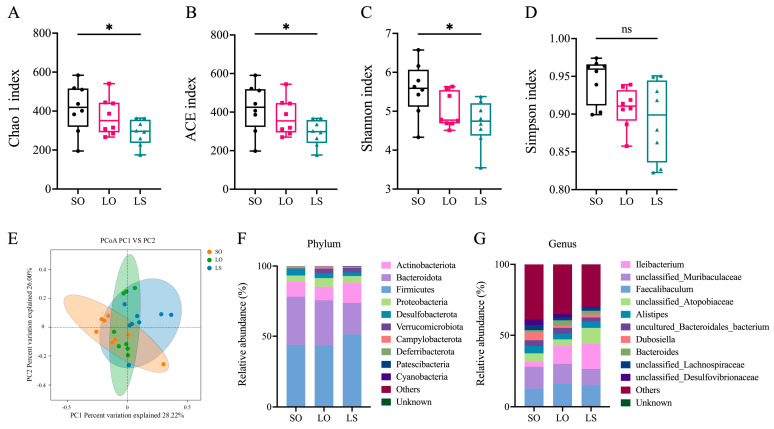
Effect of a mixture of soybean oil and lard on the gut microbiota composition. (**A**) Chao1 index; (**B**) ACE index; (**C**) Shannon index; (**D**) Simpson index of each group. (**E**) Principal coordinates analysis (PCoA) of weighted unifrac. All phyla (**F**) and genera (**G**) of gut microbiota. Data represent median (interquartile range) (*n* = 8). * *p* < 0.05 represents the significant difference.

In contrast to the SO group, the LS group displayed significantly lower values for the Chao1 index, ACE index, and Shannon index, with no significant differences observed among the remaining groups (Figure 7A–D). The principal coordinates analysis (PCoA) revealed the sample dispersion among the three groups, indicating significant differences in gut microbiota composition (Figure 7E). Additionally, *Firmicutes* were identified as the most abundant phylum across the three groups, and the genera with the highest relative abundance in the SO, LO, and LS groups were *unclassified_Muribaculaceae*, *Faecalibaculum*, and *Ileibacterium*, respectively (Figure 7F–G).

**Figure 8:**
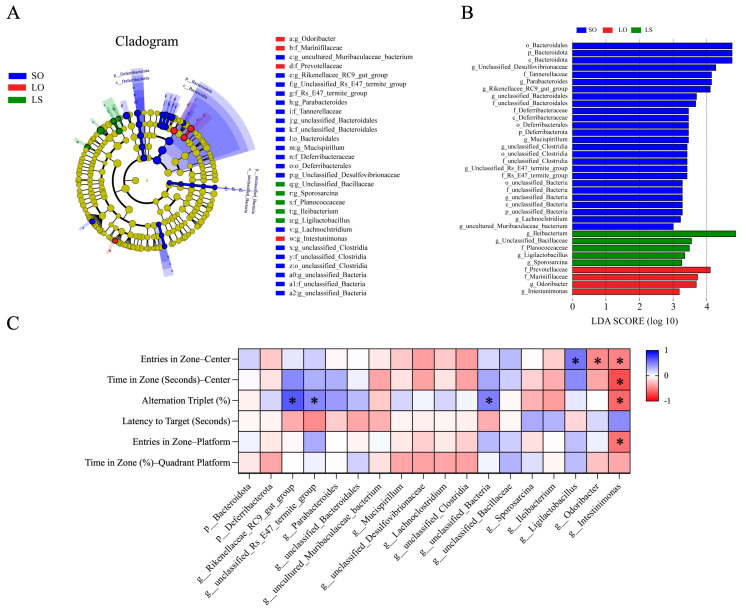
Effect of a mixture of soybean oil and lard on the gut microbiota composition. The (**A**) cladogram (LDA > 3) and (**B**) LDA score of the taxa obtained from LEfSe analysis. (**C**) The Pearson correlation analysis between behavioral testing outcome indicators and the biomarkers in microbiota from each group. * *p* < 0.05.

The LEfse analysis revealed the significantly different taxa (biomarkers) among the SO, LO, and LS groups (Figure 8A,B). At the phylum level, the relative abundance of *Bacteroidota* and *Deferribacterota* were enriched in the SO group compared with the LO and LS groups. Regarding genera, the relative abundance of the *Rikenellaceae_RC9_gut_group*, *Parabacteroides*, *Mucispirillum,* and others was enriched in the SO group compared to the LO and LS groups. The relative abundance of *unclassified_Bacillaceae*, *Sporosarcina*, *Ileibacterium*, and *Ligilactobacillus* were enriched in the LS group compared with the SO and LO groups. In addition, the relative abundance of *Odoribacter* and *Intestinimonas* was enriched in the LO group compared to the SO and LS groups. The correlation analyses between behavioral testing outcome indicators and microbiota biomarkers in each group are depicted in Figure 8C. The results indicate significant positive correlations between the relative abundance of the *Rikenellaceae_RC9_gut_group*, *unclassified_Rs_E47_termite_group*, and *unclassified_Bacteria* in the SO group and behavioral testing outcome indicators. Similarly, the relative abundance of *Ligilactobacillus* in the LS group showed a significant positive correlation with behavioral testing outcome indicators. Conversely, the relative abundance of *Odoribacter* and *Intestinimonas* in the LO group exhibited significant negative correlations with these indicators.

### 3.6. Effect of a Mixture of Soybean Oil and Lard on the SCFA and ERK(1/2)/CREB/BDNF Pathway-Related Protein Levels

**Figure 9:**
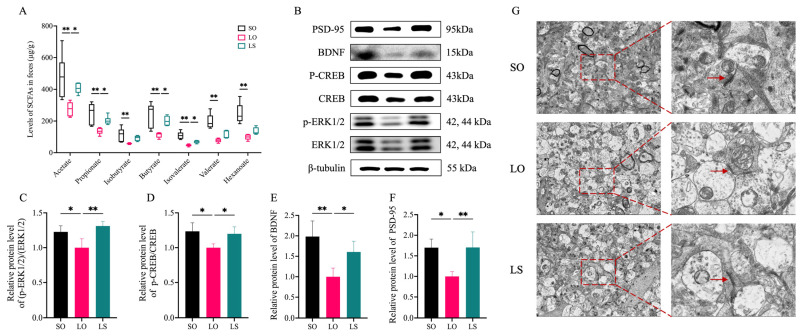
Effect of a mixture of soybean oil and lard on the SCFA and ERK(1/2)/CREB/BDNF pathway-related protein levels. (**A**) The levels of SCFAs in the feces of maternal mice (µg/g, *n* = 3). (**B**–**F**) The relative protein levels of p-ERK(1/2)/ERK(1/2), p-CREB/CREB, BDNF, and PSD-95 in brain (*n* = 4). (**G**) The ultrastructure of synapses on the transmission electron micrograph in the hippocampus (Scale bars = 2 μm and 1 μm). Red arrows: postsynaptic density. Data represent mean ± SD or median (interquartile range). * *p* < 0.05 and ** *p* < 0.01 represent the significant difference.

In comparison to both the SO and LS groups, the LO group showed significantly lower levels of acetate, propionate, butyrate, and isovalerate in feces. Specifically, levels of isobutyrate, valerate, and hexanoate were significantly lower in the LO group than in the SO group (Figure 9). It is worth noting that we observed significantly lower levels of the p-ERK(1/2)/ERK(1/2), p-CREB/CREB, and BDNF in the brains of the LO group than in those of the SO and LS groups. Interestingly, the ultrastructure of synapses on the transmission electron micrograph in the hippocampus of each group revealed that the thickness and width of postsynaptic density were lower in the LO group than in the SO and LS groups. Consistently, the postsynaptic density protein-95 (PSD-95) levels in the hippocampus of the LO group was significantly lower compared with both the SO and LS groups.

## 4. Discussion

In our study, we investigated the impact of SO, LO, and LS interventions during the pre-pregnancy to postpartum period on the cognition in an animal model. Our results show that LS exhibited a preventive effect against the postpartum cognitive impairment than LO, which was associated with modifying the brain fatty acid composition and ameliorating brain neuroinflammation, repairing gut microbiota, and upregulating the expression of the SCFA/ERK(1/2)/CREB/BDNF pathway. This provides new insights for postpartum mothers to correctly consume lard.

Fatty acids and their derivatives play various roles in the nervous system, influencing both structure and function. Among them, SFAs and MUFAs can be synthesized endogenously within the brain, which provides a degree of independence from dietary intake. However, PUFAs are predominantly acquired from the bloodstream, highlighting the importance of diet in supplying these critical nutrients [21]. Consistently, extensive studies have confirmed that dietary lipids were closely associated with brain fatty acid composition and cognitive function [22,23]. In our study, the maternal mice in the LS group demonstrated significantly better cognitive abilities than those in the LO group. This result is consistent with the differential the levels of n-3 PUFA and DHA in the maternal mice brain among the groups in our study. Previous studies have also shown that reduced levels of DHA in the brain were related to the cognitive decline, supporting the notion that adequate DHA is crucial for maintaining cognitive health [24,25]. In addition, the significant positive correlations were observed between the DHA and total *n*-3 PUFA in the brain and behavioral testing outcome indicators. This is similar to prior research indicating that peony seed oil and fish oil increased the proportions of DHA in brain, and increased DHA in mice brain was significantly positively correlated with the results of the Y-maze, Morris water maze, and novel-object tests [20]. Our findings suggest that dietary interventions that enhance brain DHA levels may offer a promising strategy for mitigating postpartum cognitive decline. Based on these results, we further investigated the potential mechanisms underlying the protective effect of elevated DHA levels in the brain on postpartum cognitive function in the LS group.

Neuroinflammation is a key factor contributing to postpartum cognitive impairment, characterized by neuronal loss, increased inflammatory cytokine expression, and activation of glial cells [26,27]. Studies have indicated that DHA and its derivatives exhibited significant anti-inflammatory effects in extracerebral tissues [20,28]. And epidemiological studies have further demonstrated that higher dietary intake of DHA is associated with a reduced incidence of neurodegenerative and neuropsychiatric conditions marked by inflammatory processes, such as Alzheimer’s disease and depressive disorders [29]. This association has prompted the hypothesis that DHA may exert similar anti-inflammatory actions within the central nervous system. Indeed, it was observed that maternal mice in the LS group exhibited fewer neuronal damages, significantly reduced levels of glial cell activation, and significantly lower levels of IL-6, TNF-α, IL-1β, and IL-18, compared with the LO group in our study. These observations are consistent with prior research, which highlighted significant morphological and functional changes in glial cells during pregnancy and the postpartum period. These changes were associated with anxiety and show a high sensitivity to variations in dietary oil intake [30]. Microglia are considered as the primary innate immune cells within the central nervous system, responding initially to pathological disturbances [31]. Concurrently, astrocytes, which represent the most populous glial cell type in the brain, play critical roles in regulating inflammatory responses, preserving the integrity of the blood–brain barrier, modulating synaptic activity, and facilitating the clearance of apoptotic cells [32]. The anti-inflammatory properties of DHA may be attributable to its modulatory impact on glial cell activity [33,34]. Indeed, a previous study has demonstrated that insufficient intake of *n*-3 PUFAs induced activation of microglia and the subsequent generation of pro-inflammatory cytokines within the hippocampus of the mice [35]. To further investigate the molecular mechanisms underlying glial cell activation, we quantified the levels of NLRP3 inflammasome complex-related proteins. The NLRP3 inflammasome complex is considered a possible therapeutic target for preventing various cognitive impairments due to its response to microbial infections and environmental stimuli [36], and it can activate caspase-1 and promote the maturation of IL-1β and IL-18 [37]. Importantly, it has been indicated that fish oil enriched with *n*-3 PUFAs mitigated postpartum depression by inhibiting the NLRP3 inflammasome-driven inflammatory pathway in an animal model [38]. Taken together, the maternal mice from the LS group exhibited significantly superior cognitive functions than the LO group, which may be due to a reduction in neuroinflammatory levels, mediated by DHA through the inhibition of inflammasome activation in the brain.

The gut microbiota significantly influence the host through various pathways, including neurological, immunological, neuroendocrine, and metabolic processes, all of which are implicated in the development of numerous neuropsychiatric disorders [39,40]. It has been established that the microbiota composition of mice with postpartum depression differed from that of healthy individuals [41]. Furthermore, it has been demonstrated that fecal transplantation from aged APP/PS1 transgenic mice into young mice resulted in a significant decline in cognitive function and showed prominent Alzheimer’s disease pathological characteristics [42]. Given these insights, we evaluated the effect of soybean oil, lard, and the mixture of soybean oil and lard on gut microbiota composition, as a potential mechanism underlying behavioral alterations. Our study reveals significant alterations in gut microbiota composition and various biomarkers among the three dietary intervention groups. The relative abundance of the *Rikenellaceae_RC9_gut_group* in the SO group screened out by LEfSe analysis showed a significant positive correlation with the behavioral testing outcome indicators. Additionally, the relative abundance of *Ligilactobacillus* in the LS group exhibited a significant positive correlation with the outcome indicators of behavioral assessments. These observations align with previous research indicating that the amelioration of anxiety- and depression-related behaviors may be associated with an increased relative abundance of the *Rikenellaceae_RC9_gut_group*, consequently attenuating xanthine oxidase activity within the brain [43]. However, research on the impact of this genus on cognitive function is relatively scarce, necessitating in-depth investigation in the future. *Lactobacillus* has been reclassified into 25 distinct genera, encompassing the *Lactobacillus delbrueckii group*, *Paralactobacillus*, and 23 novel genera. *Ligilactobacillus* represents one of the newly designated genera, constituting a subgroup specifically adapted to varied ecological environments [44]. Previous studies have revealed that the relative abundance of the *Ligilactobacillus* genus was significantly higher in the treatment group compared with the cognitive dysfunction group, leading researchers to hypothesize that the mechanism for cognitive improvement may be via the microbiota–gut–brain axis [45,46]. Notably, both the *Rikenellaceae_RC9_gut_group* and *Ligilactobacillus* are recognized producers of short-chain fatty acids (SCFAs). For example, the *Rikenellaceae_RC9_gut_group* has been positively correlated with levels of acetic acid and isobutyric acid in the gut of mice [47]. Similarly, an increase in the abundance of *Ligilactobacillus* was concomitant with elevated levels of acetic acid, propionic acid, and butyric acid in the mice gut. Thus, we conducted Western blotting to further investigate the molecular mechanisms underlying the protection of postpartum cognitive function by SCFAs.

SCFAs are recognized as crucial mediators between the gut and brain, and unraveling the signaling pathways that connect SCFAs to brain functions could pave the way for new avenues of research [48]. This suggests that the level of SCFA production may have an impact on postpartum cognitive function. We observed significantly lower levels of acetate, propionate, butyrate, and isovalerate in the LO group compared with the LS group, but the behavioral testing results of the two groups of maternal mice were opposite. Previous research indicated that improving postpartum depressive-like behaviors may have resulted from the reconstitution of the gut microbiota composition and the elevation of the SCFA levels, including acetate, propionate, butyrate, and isovalerate [41]. A recent study further supported the notion that the elevated levels of fecal SCFAs and the amelioration of gut dysbiosis through an increased abundance of SCFA-producing bacteria may mitigate depression-like and anxiety-like behaviors in an animal model of postpartum depression [49]. Furthermore, SCFAs have been shown to activate the cAMP signaling pathway [15,16], and ERK1/2, CREB, and BDNF are pivotal nodes of this signaling pathway. ERK 1/2, a constituent of the mitogen-activated protein kinase (MAPK) pathway, plays a role in the cholinergic system’s modulation of learning and memory processes [50] and is also implicated in synaptic plasticity and the consolidation of long-term memory [51]. CREB, a downstream effector of ERK, is a critical molecular substrate for learning and memory. The gene expression promoted by CREB activation is a necessary condition for establishing long-term memory and regulating synaptic plasticity [52]. BDNF, as part of the neurotrophin family, impacts neuronal growth and development. Activation of the ERK(1/2)/CREB pathway initiates the transcription of BDNF, culminating in its upregulation [53]. In our study, we found that the relative levels of p-ERK(1/2)/ERK(1/2), p-CREB/CREB, and BDNF in the brains of the LO group were significantly lower than those in the LS group. A prior study has revealed that the elevation of BDNF expression via the ERK/CREB signaling pathway in the hippocampus correlated with increased sucrose preference, locomotor activities, and a reduction in the immobility duration in the water for a mouse model of postpartum depression [54]. Moreover, it has been reported that the neuroprotective effects, which involve the inhibition of glial activation and reduction in inflammatory cytokine production, may be regulated by the ERK/CREB/BDNF/TrkB pathway [18]. PSD-95 is a critical postsynaptic density molecule that plays a pivotal role in synaptic ultrastructure and contributes to activity-dependent synaptic plasticity [55]. And the expression of BDNF protein has a direct regulatory effect on synaptic density and synaptic growth [56]. Therefore, the significantly lower PSD-95 content in the LO group compared to the LS group may partially explain why it can prevent postpartum cognitive impairment.

## 5. Conclusions

This study indicates that consuming a mixture of soybean oil and lard in a 1:1 ratio during the pre-pregnancy to postpartum period has a protective effect against postpartum cognitive impairment in maternal mice compared with lard alone, which is probably due to the alternation of brain fatty acid composition, mitigation of neuroinflammation, restoration of the gut microbiota, and upregulated expression of the SCFA/ERK(1/2)/CREB/BDNF pathway. Collectively, these mechanisms contribute to the observed enhancement in cognitive function, indicating that the soybean oil and lard mixture could serve as a promising dietary intervention for mitigating postpartum cognitive deficits.

## Figures and Tables

**Table 1:** The composition of fatty acids in the maternal mice brain (%, *n* = 6).

	SO		LO		LS	
	Mean	SD	Mean	SD	Mean	SD
C4:0	—	—	0.21	0.44	—	—
C6:0	0.48	0.72	0.52	0.91	—	—
C8:0	0.14	0.20	0.03	0.05	0.35	0.32
C10:0	0.07	0.07	0.03	0.05	0.11	0.24
C11:0	0.09	0.20	0.09	0.19	—	—
C12:0	0.01	0.01	—	—	0.02	0.03
C13:0	0.16	0.35	—	—	—	—
C14:0	0.05	0.07	0.73	0.46	0.09	0.13
C15:0	0.08	0.11	1.04	2.15	0.89	1.80
C16:0	18.70	0.91	20.16	2.48	19.50	1.18
C18:0	18.34	2.28	12.61	2.55	16.26	2.14
C20:0	0.34	0.75	—	—	0.06	0.13
C23:0	3.13	0.74	1.24	0.91	2.55	0.52
SFA	41.56	2.07	36.64	5.06	39.81	2.76
C14:1	—	—	0.30	0.41	—	—
C15:1	3.02	1.33	4.19	2.29	3.39	0.61
C17:1	—	—	0.33	0.68	—	—
C18:1 (C)	25.27	3.17	34.39	9.32	27.76	6.51
C20:1	—	—	0.27	0.57	—	—
C22:1	—	—	—	—	0.03	0.07
MUFA	28.28	3.01	39.48	8.18	31.18	6.10
C18:3 (*n*-3)	1.25	1.15	0.56	0.55	0.85	0.41
C20:3 (*n*-3)	—	—	0.18	0.38	1.29	1.99
C20:5 (EPA)	0.06	0.13	0.19	0.38	0.03	0.06
C22:6 (DHA)	18.13	1.74	12.36	0.98	16.32	1.30
*n*-3 PUFA	19.44	1.83	13.29	1.34	18.49	2.44
C20:4	10.72	0.90	10.59	3.99	10.52	1.68
*n*-6 PUFA	10.72	0.90	10.59	3.99	10.52	1.68
*n*-3/*n*-6	1.82	0.09	1.47	0.51	1.77	0.14

“—” means not detected.

## Data Availability

The original data presented in the study are openly available in FigShare at https://doi.org/10.6084/m9.figshare.25913854.v2 (accessed on 1 August 2024) and https://doi.org/10.6084/m9.figshare.25913812.v2 (accessed on 1 August 2024).

## Supplementary Materials

The following supporting information can be downloaded at https://www.mdpi.com/article/10.3390/nu16162641/s1, Table S1: Nutrition ingredients of standard diet (g/kg); Table S2: The composition of fatty acid in the standard diet.
