# Supplementary material for: A Mixture of Soybean Oil and Lard Alleviates Postpartum Cognitive Impairment via Regulating the Brain Fatty Acid Composition and SCFA/ERK(1/2)/CREB/BDNF Pathway
Source: Nutrients. 2024 Aug 10;16(16):2641. doi: 10.3390/nu16162641 (PMC11357458; doi:10.3390/nu16162641)
Supplement: Supplementary file 1 [file nutrients-16-02641-s001.zip › nutrients-3106797-supplementary.pdf]

Table S1. Nutrition ingredients of standard diet (g/kg).

Ingredient	SO	LO	LS
Casein	200	200	200
L-Cystine	3	3	3
Corn starch	397	397	397
Maltodextrin 10	132	132	132
Sucrose	100	100	100
Cellulose	50	50	50
Soybean oil	70	0	35
Lard oil	0	70	35
T-butylhydroquinone	0.014	0.014	0.014
Mineral mix SA0022M	35	35	35
Vitamin mix V1OO37	10	10	10
Choline bitartrate	2.5	2.5	2.5
Total energy (1000 g)	3850	3850	3850
Energy from protein (%)	20.3	20.3	20.3
Energy from carbohydrate (%)	63.9	63.9	63.9
Energy from fat (%)	15.8	15.8	15.8

SO, soybean oil; LO, lard oil; LS, mixed oil of lard oil and soybean oil at the ratio of 1:1.

Table S2. The composition of fatty acid in the standard diet.

	SO	LO	LS
C14:0	—[1]	1.44	0.66
C16:0	10.67	21.54	14.71
C16:1	0.00	3.13	1.75
C18:0	3.01	7.68	3.50
C18:1 (C)	24.26	45.77	34.31
C18:2 (C)	54.79	19.35	39.62
C18:3 (n-3)	7.26	1.10	5.44
SFA	13.68	30.66	18.87
MUFA	24.26	48.89	36.06
PUFA	62.06	20.45	45.07

[1] "—" means not detected. SFA, saturated fatty acid; MUFA, monounsaturated fatty acid; PUFA, polyunsaturated fatty acid. SO, soybean oil; LO, lard oil; LS, mixed oil of lard oil and soybean oil at the ratio of 1:1.
